# CT venography correlate of transverse sinus stenosis and venous transstenotic pressure gradient in unilateral pulsatile tinnitus patients with sigmoid sinus wall anomalies

**DOI:** 10.1007/s00330-020-07415-2

**Published:** 2020-10-30

**Authors:** Pengfei Zhao, Heyu Ding, Han Lv, Xiaoshuai Li, Xiaoyu Qiu, Rong Zeng, Guopeng Wang, Jian Wei, Long Jin, Zhenghan Yang, Shusheng Gong, Zhenchang Wang

**Affiliations:** 1 Department of Radiology, Beijing Friendship Hospital, Capital Medical University, No. 95, Yongan Road, Xicheng District, Beijing, 100050 China; 2 Department of Otorhinolaryngology Head and Neck Surgery, Beijing Friendship Hospital, Capital Medical University, No. 95, Yongan Road, Xicheng District, 100050 Beijing, China; 3 Department of Intervention, Beijing Friendship Hospital, Capital Medical University, No. 95, Yongan Road, Xicheng District, Beijing, 100050 China

**Keywords:** Tinnitus, Transverse sinuses, Diverticulum, Tomography, X-ray computed

## Abstract

**Objectives:**

To investigate the correlation between transverse sinus stenosis (TSS) and transstenotic pressure gradient (TPG) in unilateral pulsatile tinnitus (PT) patients with sigmoid sinus wall anomalies (SSWA).

**Methods:**

Fifty-seven patients with unilateral venous PT were retrospectively included. All of them underwent CT venography and catheter manometry, accompanied with SSWA. The degree, length, shape (intrinsic/extrinsic/dysplasia), location (proximal/middle/distal, referring to the relative position of TSS and the Labbé vein junction) of TSS, the types of SSWA (dehiscence/diverticulum), and the degree of transverse sinus outflow laterality were assessed, and the correlations with ipsilesional TPG were analyzed.

**Results:**

The mean value of ipsilesional TPG was 7.61 ± 0.52 mmHg. The degree and length of ipsilesional TSS were positively correlated with TPG (*p* < 0.001, *p’* < 0.001), respectively. TPG was significantly larger in patients with contralateral transverse sinus dysplasia than those without (*p* = 0.023) and significantly smaller in patients with ipsilesional sigmoid sinus diverticulum than those with isolated dehiscence (*p* = 0.001). No statistical difference in TPG was shown between ipsilesional TSSs of different shapes or locations (*p* > 0.05). No correlation was noted between the degree of ipsilesional transverse sinus outflow laterality and TPG (*p* = 0.051). Stepwise linear regression indicated that the degree (*β* = 9.207, 95% CI = 3.558–14.856), length (*β* = 0.122, 95% CI = 0.025–0.220) of ipsilesional TSS, and contralateral transverse sinus dysplasia (*β* = 1.875, 95% CI = 0.220–3.530) were significantly correlated with TPG (*R*^2^ = 0.471).

**Conclusions:**

The degree, length of ipsilesional TSS, and contralateral transverse sinus dysplasia may be used to predict TPG in unilateral PT patients with SSWA.

**Key Points:**

• *CT venography may act as a screening tool to help low-probability unilateral pulsatile tinnitus (PT) patients with sigmoid sinus wall anomalies (SSWA) avoid invasive catheter manometry.*

• *The degree and length of ipsilesional transverse sinus stenosis (TSS) are positively correlated with transtenotic pressure gradient (TPG) in unilateral PT patients with SSWA.*

• *Ipsilesional TPG is larger in unilateral PT patients with contralateral transverse sinus dysplasia than those without and is smaller in unilateral PT patients with sigmoid sinus diverticulum than those with isolated dehiscence.*

## Introduction

Pulsatile tinnitus (PT) is an annoying pulse-synchronous sound in the ear, usually occurring unilaterally in women of childbearing age. The long-term existence of PT seriously affects patients’ quality of life and may cause both functional and structural cerebral changes [1]. The etiology of PT can be divided into arterial, venous, and others.

Sigmoid sinus wall abnormalities (SSWA) are currently recognized as the most common venous etiology [2, 3], including sigmoid sinus wall dehiscence and sigmoid sinus diverticulum [4]. These patients are often accompanied with transverse sinus stenosis (TSS) [4–7], ipsilesional venous outflow dominance [8], high jugular bulb [8], empty sella [4, 8], well-pneumatized temporal bone [8, 9], jet-like flow [10–12], and intraluminal vortex/turbulence [10, 11, 13], suggesting the occurrence of PT may be the result of a multifactorial combination, including blood vessel, blood flow, bony wall, conduction, and increased intracranial pressure.

The transtenotic pressure gradient (TPG) of transverse sinus has been reported as a key factor for PT, which plays a role in generating jet-like flow and strong impact force toward the sigmoid sinus wall [14]. The long-term impact of abnormal blood flow that is related to TPG may be responsible for the progressive erosion of bony wall of the sigmoid sinus, which results in SSWA [6]. After then, the flow sound transmits to the inner ear through the dehiscent area [15].

Transtemporal sigmoid sinus wall reconstruction has been mostly reported to treat PT with SSWA, mainly aiming to reconstruct the barrier of sound conduction and sinus wall vibration [13, 15]. Stent implantation is an alternative procedure in PT with TSS, aiming to alleviate elevated TPG and corresponding jet-like flow [7, 14]. Both treatments have been reported with good results. The higher the TPG of PT patients, the more likely it will be suitable for stent implantation, regardless of the problems caused by anticoagulant drugs.

Not all TSSs are accompanied by elevated TPG. For PT patients with TSS but without elevated TPG, stent implantation should be more cautiously employed. The accurate evaluation of TPG depends on the manometric measurement by invasive digital subtraction angiography (DSA), which may be challenging to be widely performed in PT patients due to its invasiveness. Thus, it is necessary to explore a noninvasive method to help predict TPG.

However, there has been no report about the characteristics of TPG in PT patients, as far as we can ascertain. Besides, the role of noninvasive radiologic methods in the relationship between TSS and TPG should be further evaluated. West JL et al [16] reported that the degree of TSS measured on DSA had a linear correlation with TPG, while TSS on MR venography/CT venography (CTV) had low diagnostic efficiency for TPG.

In this study, the TPG was measured using catheter manometry in unilateral venous PT patients with SSWA; the degree, length, relative location and shape of TSS, the types of SSWA, and the degree of venous outflow laterality were evaluated using CTV; the correlations between features associated with TSS and TPG were analyzed. The results may facilitate the understanding of the pathophysiology of venous PT and formulating appropriate evaluation and treatment strategies.

## Materials and methods

### Clinical data

This study was approved by the ethics committee in our institution. Informed consent was obtained from each patient. A consecutive series of 645 patients admitted to our hospital for PT from January 2017 to December 2019 were retrospectively included. The inclusion criteria were as follows: (1) unilateral noise, the rhythm was consistent with the heartbeat, and the sound disappeared or significantly decreased after the compression of ipsilesional jugular vein; (2) preoperative CTV examination; (3) manometric measurement by DSA within 3 months after CTV examination; (4) SSWA on CTV. Exclusion criteria were as follows: CT or DSA showed aneurysms, arteriovenous malformation/fistula, abnormal aberrant vessels communicating with the middle ear, and neoplastic causes.

### CT examination and manometric measurement

#### CT examination

CT images were acquired using a 64-section CT scanner (Brilliance, Philips Healthcare) or a 256-section CT scanner (Revolution, GE Healthcare). The parameters were as follows: 100 kV, auto-mAs, matrix 512 × 512, collimation 64 or 256 × 0.625 mm, rotation time 0.5–0.75 s, and threshold of trigger bolus tracking 120 HU. Contrast media (iopamidol, Bracco Diagnostics) are 370 mg iodine/mL, 1.5 mL/kg, and 5 mL/s. The arterial images were scanned along caudal-cranial direction and reconstructed with standard algorithm to evaluate the potential arterial causes of PT. The venous images used in this study were obtained along cranial-caudal direction 7 s later and reconstructed with both standard algorithm and bone algorithm.

#### Manometric measurement

DSA was performed under local anesthesia using an angiography machine (Innova 4100-iq, GE Healthcare). Right femoral artery was punctured with Seldinger’s technique to complete routine DSA. The venous sinuses including the ipsilesional TSS were observed especially. After that, the right femoral vein was punctured based on Seldinger’s technique. A 5-F catheter was intubated into the jugular vein. Then, the superior sagittal sinus was navigated using a 2.7-F coaxial microcatheter (Stride, Asahi), which was connected to a standard pressure transducer (DPT-248, Yixinda). The pressure values were measured in ipsilesional transverse sinus distal to the TSS and sigmoid sinus proximal to the TSS, respectively. The TPG was calculated as the difference value in mmHg.

### Interpretations of TSS on CTV

CT data were delivered to the postprocessing workstation (AW 4.6, GE Healthcare) for curved planer reformation. All images were reviewed independently by two experienced neuroimaging radiologists who were unknown to the clinical and manometric data. The two measurements of the same sample were averaged for the quantitative results. The discrepancies for both quantitative and qualitative results were further judged by a senior neuro-radiologist with more than 20 years of work experience.

#### The degree of TSS (Fig. 1)

Based on CT curved planer reformation, cross-sectional area of ipsilesional TSS was measured for each patient. Any sinus with multiple TSS was measured at the most severe point. Normative area was defined as the cross-sectional area of nonstenosed transverse sinus at the distal end of the stenosis, avoiding the location where cortical veins converge. The degree of TSS was calculated by dividing the area of TSS by the normative area.

**Fig. 1:**
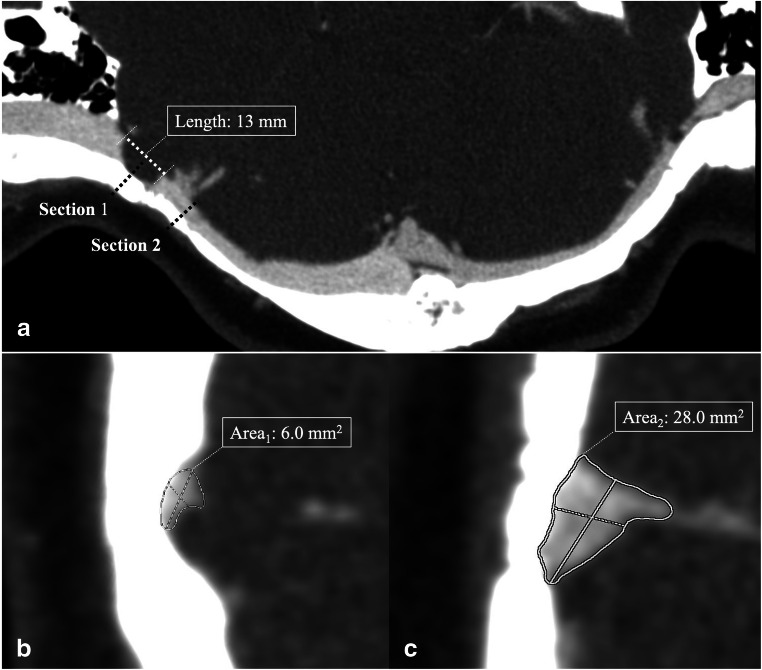
The degree and length of transverse sinus stenosis. The entire course of transverse sinus is shown in a curved reformatting CT image (**a**). The percentage of transverse sinus stenosis is calculated by dividing the cross-sectional area of stenosis (**b**) by the normative area of adjacent transverse (**c**). The range is also measured based on CT curved planer reformation

#### The length of TSS (Fig. 1)

The length of TSS was also measured based on CT curved planer reformation. Any sinus with multiple TSS was measured at the most severe one.

#### The shape of TSS (Fig. 2)

TSS was divided into different morphological forms: (1) an intrinsic stenosis was found with a focal intraluminal filling defect, mostly caused by the arachnoid granulation. (2) An extrinsic stenosis was defined as the long smooth tapered narrowing of external compression, mostly caused by brain parenchyma [17]. (3) Hypoplasia was defined as the decrease of more than 50% segment of transverse sinus, the extent of whose cross-sectional area was less than 50% of that of contralateral transverse sinus. (4) Aplasia was defined as complete flow gap. Hypoplasia and aplasia were collectively referred to as dysplasia.

#### The location of TSS (Fig. 2)

The location of TSS was defined based on the relative position of TSS and the confluence point of the Labbé vein. A proximal TSS was defined when TSS was located at the proximal end of the confluence point of the vein. A middle TSS was defined when the vein jointed into the area of TSS. A distal TSS was defined when TSS was located at the distal end of the confluence point of the vein.

**Fig. 2:**
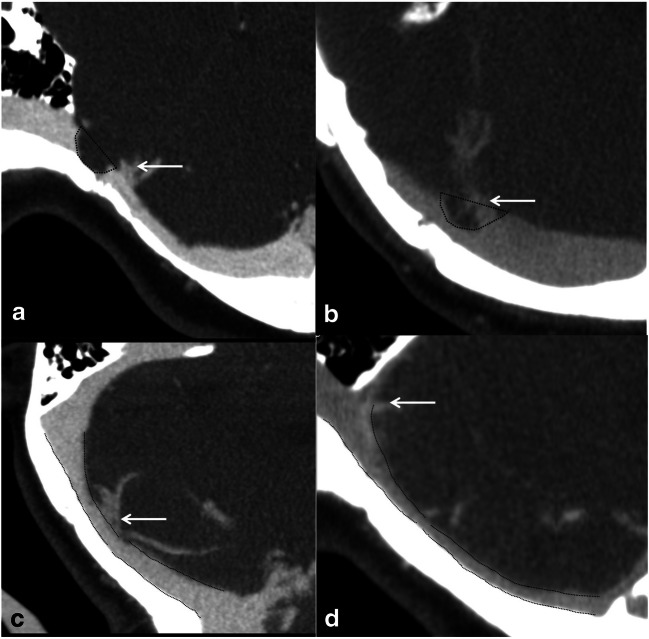
The shape and location of transverse sinus. An intrinsic stenosis is defined as a focal intraluminal filling defect (**a** and **b**), while an extrinsic stenosis is defined as the long smooth tapered narrowing of external compression (**c** and **d**). A proximal stenosis is defined when the stenosis is located at the proximal end of the Labbé vein (**a**). A middle stenosis is defined when the Labbé vein joins into the area of stenosis (**b** and **c**). A distal stenosis is defined when the stenosis is located at the distal end of the Labbé vein (**d**)

#### The degree of transverse sinus outflow laterality

The degree of transverse sinus outflow was calculated by dividing the normative cross-sectional area on the symptomatic side by the sum of both sides.

#### Sigmoid sinus wall dehiscence and diverticulum (Fig. 3)

Sigmoid sinus wall dehiscence was identified as a focal defect in the bony wall overlying the sigmoid sinus with a normal sigmoid contour in at least two consecutive axial 1-mm slices [8]. Sigmoid sinus diverticulum was identified as a focal out-pouching of the sigmoid sinus exerting into the mastoid bone or air cells [4].

**Fig. 3:**
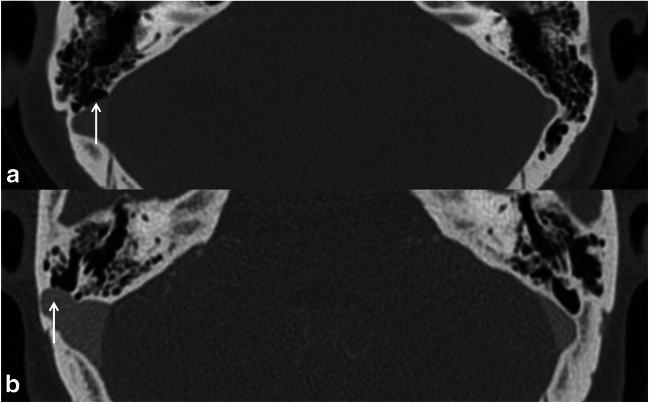
Axial high-resolution CT venography images show sigmoid sinus wall dehiscence (**a**) and sigmoid sinus diverticulum (**b**) on the right sides

### Statistical analysis

Statistical analyses were performed using SPSS (Version 22.0; SPSS). Medians (interquartile ranges) or means ± standard deviations were obtained for continuous variables. A two-tailed *p* value of 0.05 was considered statistically significant. The differences in TPG of two categories were compared using the independent-sample *t* test or Mann-Whitney *U* test. The difference in TPG of the three categories was compared using the one-way ANOVA. Unary linear regression was performed for the relationships between TPG and continuous variables. The significant variables in univariate analysis were further used to fit a multivariate linear regression model using the stepwise method.

## Results

Fifty-seven patients were included, including 50 females and 7 males. Forty-three patients were found with PT on the right side and 13 on the left side. The median age was 36 (19) years old. The mean TPG was 7.61 ± 0.52 mmHg.

The relationships between the characteristics of TSS and TPG were shown in Tables 1 and 2. The results showed that the degree, length of ipsilesional TSS, and contralateral transverse sinus dysplasia were positively correlated with TPG. PT patients with isolated sigmoid sinus wall dehiscence had a larger TPG than those with diverticulum. The shape of ipsilesional TSS, the relative position between TSS and the Labbé vein junction, and the degree of outflow dominance laterality did not show significant effect on TPG.

**Table 1:** Results of differences in transtenotic pressure gradient between groups of categorical variables

Variables	No. (%)	TPG (mmHg)	*t*	*p*
Contralateral transverse sinus dysplasia	23 (40.4)	9.02 ± 4.12	2.339	0.023
Contralateral transverse sinus without dysplasia	34 (59.6)	6.65 ± 3.47		
Intrinsic stenosis	31 (54.4)	7.35 ± 3.35	− 0.524	0.603
Extrinsic stenosis	26 (45.6)	7.91 ± 4.50		
Distal stenosis	9 (15.8)	5.51 ± 2.27		0.104^a^
Middle stenosis	27 (47.4)	7.97 ± 4.08	–	0.106^b^
Proximal stenosis	21 (36.8)	8.04 ± 4.04		0.952^c^
Sigmoid sinus wall dehiscence	34 (59.6)	8.92 ± 3.92	3.387	0.001
Sigmoid sinus diverticulum	23 (40.4)	5.66 ± 2.97		

^a^*p* value between distal stenosis group and middle stenosis group; ^b^*p* value between distal stenosis group and proximal stenosis group; ^c^*p* value between proximal stenosis group and middle stenosis group

**Table 2:** Results of differences in transtenotic pressure gradient for continuous variables

Variables	Means/medians	*β*	95% confidence interval	*R*^2^	*p*
Percentage of TSS	74.5% (15%)	13.313	7.204–19.422	0.257	< 0.001
Length of TSS	23.21 ± 9.38 mm	0.189	0.090–0.289	0.209	< 0.001
Percentage of Ipsilesional venous outflow	74.7 ± 15.8%	6.411	–	–	0.051

*TSS*, transverse sinus stenosis

After adjusting the factors including the degree, length, contralateral transverse sinus dysplasia, and the types of sigmoid sinus wall anomalies, stepwise linear regression showed that the degree (*β* = 9.207, 95% CI: 3.558–14.856), length (*β* = 0.122, 95% CI: 0.025–0.220) of ipsilesional TSS, and contralateral transverse sinus dysplasia (*β* = 1.875, 95% CI: 0.220–3.530) were correlated with TPG (*R*^2^ = 0.471). The equation was as follows: y_TPG_ = − 0.172 + 9.207*x*_percentage of TSS_ + 0.122*x*_length of TSS_ + 1.875*x*_contra-dysplasia_.

## Discussion

There have been few studies about TPG in patients with PT. Lenck S et al [14] reported that the median of ipsilesional TPG was 9 mmHg in 14 venous PT patients, including 8 patients with IIH. After stenting, all patients got complete resolution of PT and obvious decrease or even elimination of TPG. Our results showed a similar value in unilateral PT patients with SSWA, implying that the increased TPG may be an important sign of venous PT.

Different from showing the size of transverse sinus by the width measured on axial slices in most previous studies, curved planer reformation was performed to show the whole course of the transverse sinus, and the cross-sectional area was measured in this study. Considering the irregular shape of transverse sinus, especially for TSS, this method may have the potential of reflecting the size more accurately.

Semi-quantitative methods were mostly used to determine TSS previously. Width difference of more than 50% or 75% was usually used [5, 8]. A combined conduit score described by Farb et al [18] and a new index described by Carvalho GB et al [19] have also been adopted [6, 20]. Those methods may partially obscure the effect of TSS in PT and sometimes make it difficult to compare the results. Thus, the percentage of TSS was calculated and used in this study.

West JL et al [16] stated that an increase in TPG of 3.5 mmHg was seen for every 10% increase in TSS in IIH patients. Our results also showed a linear correlation between TSS and TPG in PT patients, while the degree of TSS had a much smaller effect on TPG. The discrepancy may be related to the differences in the subjects included and measurement methods. Besides, we found for the first time that the greater the length of TSS, the larger the TPG. This result may be partly explained by the fact that as TSS lengthens, the resistance required to pass through the stenosed transverse sinus increases.

Dinkin M et al [21] proposed that compared with the degree of TSS, the shape of TSS may have a more important impact on the clinical course of IIH patients. Lasley JA et al [5] speculated that the association may exist between the shape of TSS and PT. Thus, we evaluated the relationship between the shape of TSS and TPG, but the results did not show a significant difference in TPG between the intrinsic stenosis group and extrinsic stenosis group. However, ipsilesional TPG was found significantly larger in patients with contralateral transverse sinus dysplasia, suggesting that TPG may be related to the dominant blood flow and alternative pathways. Considering the possible effect of blood flow on TPG, we further quantified the degree of ipsilesional blood flow dominance. The results did not show a clear correlation between the proportion of ipsilesional transverse sinus area and TPG. It should be pointed out that the size of the sinus cavity does not fully represent the magnitude of blood flow, which needs to be further evaluated using hemodynamic methods.

Considering the possible influence of alternative pathways on TPG, we evaluated the relative position relationship between TSS and cortical veins that flow into the transverse sinus. The cortical veins that flow into the transverse sinus are mainly Labbé veins, along with the venules from the tentorial area of the cerebellum. Our results showed that TPG was relatively small when Labbé veins merged into the proximal side of TSS with lower pressure. However, there was no significant difference when compared with the middle and proximal TSSs, respectively. Whether the relative position of collateral veins and TSS has an impact on TPG needs to be further studied.

It is very interesting to find that TPG of patients with isolated sigmoid sinus wall dehiscence was larger than that of patients with sigmoid sinus diverticulum. This result showed that the diverticulum may require a relatively smaller pressure gradient to generate PT than isolated sigmoid sinus wall dehiscence, which may be explained by the fact that the diverticulum itself may generate or aggravate the turbulence of flow for PT [4].

This study has several limitations. Firstly, this research lacked a control group to assess the relationship between TSS and TPG in people without PT. We assume that they may share a similar pattern, which is however difficult to be obtained in asymptomatic population because of the invasiveness of TPG measurement. This speculation may be further judged in IIH patients. Secondly, we used the morphological data of TSS to reflect the characteristics of blood flow, and the speculation of blood flow needs to be verified*.* We presume that the hemodynamic data of transverse sinus (such as transstenotic velocity gradient, transstenotic flow gradient) may show more significant correlations with TPG than the morphological data of transverse sinus, which should be evaluated using noninvasive methods, such as 4D flow MR, computational fluid dynamics, or both. In addition, lumbar puncture had not been performed in most of the patients above, and the relationship among TSS, TPG, and intracranial pressure should be further specially investigated.

In summary, noninvasive CTV may serve as a screening tool to help low-probability patients avoid catheter manometry for TPG and stent implantation. The degree, length of ipsilesional TSS, and contralateral transverse sinus dysplasia may be used to predict TPG. Besides, unilateral PT patients with isolated sigmoid sinus wall dehiscence may have a larger TPG than those with diverticulum. These results may contribute to understand the pathophysiological process of venous PT.

## Abbreviations

CI: Confidence interval
CTV: CT venography
DSA: Digital subtraction angiography
PT: Pulsatile tinnitus
SSWA: Sigmoid sinus wall anomalies
TPG: Transtenotic pressure gradient
TSS: Transverse sinus stenosis
